# *Enterococcus faecium* HDRsEf1 Protects the Intestinal Epithelium and Attenuates ETEC-Induced IL-8 Secretion in Enterocytes

**DOI:** 10.1155/2016/7474306

**Published:** 2016-11-07

**Authors:** Zhongyuan Tian, Xiaofang Liu, Ran Dai, Yuncai Xiao, Xiliang Wang, Dingren Bi, Deshi Shi

**Affiliations:** ^1^State Key Laboratory of Agricultural Microbiology, College of Veterinary Medicine, Huazhong Agricultural University, Wuhan 430070, China; ^2^Key Laboratory of Development of Veterinary Diagnostic Products, Ministry of Agriculture, Huazhong Agricultural University, Wuhan, Hubei 430070, China; ^3^College of Animal Science and Technology, Agricultural University of Hebei, Baoding 071000, China

## Abstract

The probiotic* Enterococcus faecium* HDRsEf1 (Ef1) has been shown to have positive effects on piglet diarrhoea, but the mechanism has not yet been elucidated. In this study, using the IPEC-J2 cell line to mimic intestinal epithelial cells and enterotoxigenic* Escherichia coli* (ETEC) K88ac as a representative intestinal pathogen, the mechanism underlying Ef1 protection against an enteropathogen was investigated. The results demonstrated that Ef1 was effective in displacing K88ac from the IPEC-J2 cell layer. Moreover, Ef1 and its cell-free supernatant (S-Ef1) modulate IL-8 released by IPEC-J2 cells. Ef1 and its cell-free supernatant showed the potential to protect enterocytes from an acute inflammatory response. In addition, Ef1 and its cell-free supernatant increased the transepithelial electrical resistance (TEER) of the enterocyte monolayer, thus strengthening the intestinal barrier against ETEC. These results may contribute to the development of therapeutic interventions using Ef1 in intestinal disorders of piglets.

## 1. Introduction

Probiotic bacteria have long been used to promote the production of various animals and to protect the animals against pathogens, especially enteric pathogens [1, 2]. According to the World Health Organisation, probiotics are defined as live organisms that, if ingested in sufficient amounts, have beneficial effects on the overall health of the host [3]. Adhesion is considered a crucial step for intestinal bacteria to colonise and further interact with the host epithelium and immune system. Intestinal bacteria can adhere to mucus or bind to exposed intestinal epithelium cells (IECs) via their surface structures [4–7]. Porcine ETEC strains are characterised by their production of specific adhesins and enterotoxins. Fimbrial adhesin K88 (F4) and heat-stable (ST) and heat-labile (LT) enterotoxins have been identified as important factors contributing to diarrhoeal diseases [8, 9]. The swine industry has relied largely on prophylactic use of antibiotics to control ETEC and related diarrhoea. There is growing concern about the widespread of antibiotic resistance in zoonotic bacterial pathogens, which pose a threat to public health. Thus, strategies other than the use of antibiotics to control pathogens are urgently needed for swine production. In stable conditions, IECs create a tolerogenic environment, but during a pathogen infection, they release proinflammatory molecules to recruit immune cells and induce an acute inflammatory response. Inflammation is an essential physiological response to infection, but dysregulated immune responses to bacterium-derived molecules in healthy intestines can result in excessive mucosal inflammation [10]. Newborn piglet intestines are immature, and an inflammatory response may contribute to both anatomical and functional intestinal disorders [11, 12]. Interleukin-8 (IL-8) is one of the key chemokines responsible for the initiation of inflammatory cascades and recruitment of neutrophils into the mucosa [13]. Cell wall components from Gram-negative bacteria, such as lipopolysaccharides, as well as host-derived cytokines such as IL-1*β* and TNF-*α*, increase IL-8 secretion from IECs through activation of mitogen activated protein kinase (MAPK) [14, 15]. After acute inflammation, commensal bacteria are believed to play a key role in providing regulatory immune stimuli to return mediators to basal levels [1]. Recent studies also suggest that some probiotics can suppress mucosal inflammation in the gut [16–18]. The probiotic* Enterococcus faecium* HDRsEf1 strain, which was isolated by our research group, has been granted a patent in China [19] and is already being used as a feed additive for piglets. Feeding results demonstrated that HDRsEf1 could reduce the incidence and severity of diarrhoea in weaning piglets [20], and* in vitro* study in HT-29 cells suggested that HDRsEf1 may act as an antagonist to intestine inflammation response to intestine pathogen [21]. In this study, we examined the ability of HDRsEf1 to protect the integrity of IECs* in vitro* and explored whether HDRsEf1 could regulate IL-8 released by IECs.

## 2. Methods and Materials

### 2.1. Bacteria Strains and Culture Conditions

*Enterococcus faecium* HDRsEf1 (Ef1) was isolated and identified by the Department of Veterinary Microorganisms & Immunity, Huazhong Agricultural University [22]. Ef1 was cultivated in MRS medium (Qingdao Hope Bio-Technology Co., Ltd., China) for 18 h at 37°C. The subculture of the bacterium was grown 8 h and centrifuged, and then the bacterial cells (Ef1) and their cell-free supernatant (S-Ef1) were collected. Cell pellets were washed thrice in phosphate-buffered saline (1x PBS, pH 7.4). ETEC K88ac was kindly provided by Professor Jian Peng (Huazhong Agricultural University, China) and cultivated in tryptic soy broth (TSB; Becton, Dickinson and Company, San Jose, CA). The K88ac strain was incubated overnight at 37°C. A subculture of the bacterium was grown for 3 h to 4 h, until the midlog phase, and then centrifuged. Cell pellets were washed thrice in 1x PBS. Ef1 and K88ac were resuspended in antibiotic-free DMEM/F12 medium prior to experiments with IPEC-J2 cells (HyClone, Beijing, China).

### 2.2. Preparation of Ef1 Cell-Free Culture Supernatant

The cell-free supernatant from overnight cultures of Ef1 (S-Ef1) was prepared by centrifugation at 8000 rpm for 10 min at 4°C, followed by filtration through a 0.22 *μ*m filter to remove any remaining bacteria. Cell-free supernatant equivalent to 1 × 10^8^ CFU/mL was added to 1 mL antibiotic DMEM/F12 for the experiments described below.

### 2.3. Isolation and Purification of Exopolysaccharides (EPS) from S-Ef1

The EPS produced by HDRsEf1 were purified according to a procedure previously reported by Pan and Mei, with minor modifications [24]. Briefly, the proteins in the EPS broth were removed with 7.0% (v/v) trichloroacetic acid (TCA) and centrifugation at 10,000 rpm for 20 min at 4°C, and the EPS in the supernatant were precipitated from the broth by adding cold ethanol to 75% (v/v) and leaving the broth overnight at 4°C. The final precipitate was collected by centrifugation at 10,000 rpm for 20 min at 4°C and was redissolved in distilled water and then dialyzed through dialysis membrane (MW: 12000–14000, Thermo, USA) using distilled water for 24 h at 4°C. The dialyzed solution, at a concentration equivalent to the 5 × 10^7^ CFU/mL of Ef1, was added to 1 mL antibiotic-supplemented DMEM/F12 for the experiments described below.

### 2.4. Isolation and Purification of Protein from S-Ef1

The protein produced by Ef1 was purified according to a procedure previously reported by Claes et al. with minor modifications [25]. Briefly, bacteria were grown overnight in MRS medium. After centrifugation at 10000 rpm/min for 20 min, proteins were precipitated from the supernatant by incubation at 4°C for 30 min in the presence of TCA (20% final concentration). After centrifugation at 12,000 rpm for 20 min, the precipitated proteins were washed twice with cold acetone. The pellet was air dried and resuspended in DMEM/F12 and, at a concentration equivalent to the 5 × 10^7^ CFU/mL of Ef1, was added to 1 mL antibiotic-supplemented DMEM/F12 for the experiments described below.

### 2.5. Cells and Culture Conditions

Porcine epithelial cells from the jejunum (IPEC-J2) were kindly donated by Professor Li Zili (Huazhong Agricultural University). The IPEC-J2 cells were seeded in cell culture flasks and cultured in DMEM/F12 medium supplemented with 10% foetal bovine serum (FBS, Gibco, Australia), 1% penicillin-streptomycin (Sigma, USA), and 1% glutamine (Gibco, USA) at 37°C in a humidified atmosphere of 5% CO_2_ (Selecta, Barcelona, Spain). The cells were cultured for at least 10 days, with the culture medium changed every other day.

### 2.6. Adhesion and Adhesion Inhibition Assays

Approximately 5 × 10^5^ cells/mL were seeded into a 12-well plate and were cultured to allow differentiation. Adhesion assays were performed using fully differentiated IPEC-J2 cells (10 d postconfluence cultures). Bacteria were suspended in DMEM/F12 without antibiotics at concentrations of 5 × 10^7^ CFU/mL (Ef1) and 5 × 10^7^ CFU/mL (K88ac), and after the culture medium of IPEC-J2 was suck out, fresh medium containing the bacteria was added to wells and incubated for 1 h at 37°C in a 5% CO_2_ atmosphere. In the competition assay, Ef1 or S-Ef1 was added simultaneously with K88ac. For the exclusion assay, Ef1 or S-Ef1 was added first, and then 1 h later, K88ac was added and incubated for 1 h. For the displacement assay, K88ac was added first, and then 1 h later, Ef1 or S-Ef1 was added and incubated for 1 h. After incubation, nonadherent bacteria were discarded by washing thrice with sterile 1x PBS. The cells with adherent bacteria were lysed with 1 mL/well of Triton X (final concentration 1% in 1x PBS, v/v) for 10 min in an ice-water bath. K88ac adhering to IPEC-J2 cells was serially diluted and spread onto MacConkey agar medium (Qingdao Hope Bio-Technology Co., Ltd., China) for counting; Ef1 was also serially diluted and spread onto MRS to count the adherent bacteria. All experiments were performed three times independently.

### 2.7. Transepithelial Electric Resistance (TEER) Measurement

IPEC-J2 cells were seeded onto 4.2 cm^2^ Transwell®-COL collagen-coated membrane filters (24-mm pore size, Corning, USA) to polarise the monolayer. IPEC-J2 cells were seeded at 1 × 10^6^ cells per Transwell filter in 6-well tissue culture plates. TEER was measured every day after seeding, using the Millicell electrical resistance system (Millipore, Darmstadt, Germany). In order to avoid cell division, a high seed density was used to saturate the available area. At each measurement, duplicate values for at least two areas in each filter were obtained, and the results were expressed as Ω cm^2^. Cell monolayers with TEER levels above 4000 Ω cm^2^ were assumed to be fully polarised and were selected for the TEER test [26]. Into a fully polarised IPEC-J2 monolayer, 1 mL/well of Ef1 (1 × 10^8^ CFU/mL) or S-Ef1 was added, preincubated for 2 h, and then washed with sterile 1x PBS (pH 7.4) thrice. Following this, 1 mL/well of K88ac (1 × 10^8^ CFU/mL) was added as a stimulant for 12 h, and TEER of each sample was measured every 3 h. All experiments were performed three times independently.

### 2.8. Stimulation of IPEC-J2 Cells

#### 2.8.1. Pretreatment with Ef1 or S-Ef1

IPEC-J2 cells (10^5^) were seeded into 12-well plates (Corning, USA) and cultured at 37°C for 3 days in 5% CO_2_, and the cells were 100% confluent, and they were washed with sterile 1x PBS thrice, incubated with 5 × 10^7^ CFU/well Ef1 or S-Ef1 for 2 h, and washed with sterile 1x PBS thrice. Then, 1 mL/well of K88ac (5 × 10^7^ CFU/mL), 1 mL/well of IL-1*β* (2 ng/mL, 4 ng/mL, or 8 ng/mL), and 1 mL/well of TNF-*α* (50 ng/mL, 100 ng/mL, or 200 ng/mL) were added to each well and incubated for 2 h or 4 h. The bacteria, S-Ef1, IL-1*β*, and TNF-*α* were added in DMEM to IPEC-J2 cells.

#### 2.8.2. Pretreatment with Heat-Inactivated Ef1 or S-Ef1

IPEC-J2 cells (10^5^ cells/well) were seeded into 12-well plates (Corning, USA) and cultured at 37°C for 3 days in 5% CO_2_, and the cells were 100% confluent and differentiated, and they were washed with sterile 1x PBS thrice. The washed cells were treated with 5 × 10^7^ CFU/well Ef1 or S-Ef1 (heat-inactivated at 95°C for 30 min) for 2 h and washed with sterile 1x PBS thrice, and then 1 mL/well of K88ac (5 × 10^7^ CFU/mL) was added and incubated for 2 h.

#### 2.8.3. Pretreatment with EPS or Protein from S-Ef1

IPEC-J2 cells (10^5^ cells/well) were seeded into 12-well plates (Corning, USA) and cultured at 37°C for 3 days in 5% CO_2_, and the cells were 100% confluent and differentiated, and they were washed with sterile 1x PBS thrice. The washed cells per well were treated with EPS or protein equivalent to culture volume containing 5 × 10^7^ CFU Ef1 for 2 h and washed with sterile 1x PBS thrice, and then 1 mL/well of K88ac (5 × 10^7^ CFU/mL), IL-1*β* (8 ng/mL), or TNF-*α* (200 ng/mL) was added and incubated for 2 h.

### 2.9. Extraction of Total RNA and Synthesis of cDNA

After the treatment described in Section 2.6, IPEC-J2 cells were harvested and washed thrice with ice-cold 1x PBS. Total RNA from IPEC-J2 cells was extracted with a RNATM.iso PLUS Kit (Takara Biotechnology, Dalian, China). Reverse transcription (RT) was performed using a RevertAid First Strand cDNA Synthesis Kit (Takara Biotechnology, Dalian, China) according to the manufacturer's instructions.

### 2.10. Quantitative Real-Time PCR of IL-8 Transcripts

The mRNA level of IL-8 in IPEC-J2 cells described in Section 2.8 was analysed by quantitative real-time PCR (qRT-PCR). qRT-PCR was performed using SYBR Premix EX Taq (TransGen Biotech, China). Amplification was carried out in a total volume of 20 *μ*L, containing 2 *μ*L of cDNA, 10 *μ*L of SYBR Premix EX Taq, 7.2 *μ*L double-distilled H_2_O, and 0.4 *μ*L of each primer (Table 1). The amplification reactions were performed under the following PCR conditions: (i) one cycle at 95°C for 30 s and (ii) amplification with 40 cycles of 95°C for 10 s and 60°C for 20 s, followed by (iii) 95°C for 30 s, 55°C for 1 min, and 95°C for 30 s. All experiments were performed three times independently, and the data are presented as mean values obtained from three independent experiments.

### 2.11. Enzyme-Linked Immunosorbent Assay of IL-8

As described in Section 2.8, after being treated with K88ac, IL-1*β*, or TNF-*α* for 4 h, the supernatant of IPEC-J2 cells was harvested and the IL-8 level in the supernatant was measured by an IL-8 ELISA Kit, according to the manufacturer's instructions (4A Biotech Co. Ltd. ELISA Kit, Swine IL-8). All experiments were performed three times independently.

### 2.12. Statistical Analysis

Statistical evaluations were performed using the IBM SPSS-Statistics program for Windows, version 22 (International Business Machines Corp., Armonk, United States of America). Graphs were plotted with GraphPad Prism 5 software (Graphpad Software Inc., San Diego, CA). Results are given as means ± SEM. The significance level for all analyses were set to *p* < 0.05 (*∗*), *p* < 0.01 (*∗∗*), and *p* < 0.001 (*∗∗∗*). All experiments were performed three times.

## 3. Results

### 3.1. Adhesion and Adhesion Inhibition Assays

**Figure 1:**
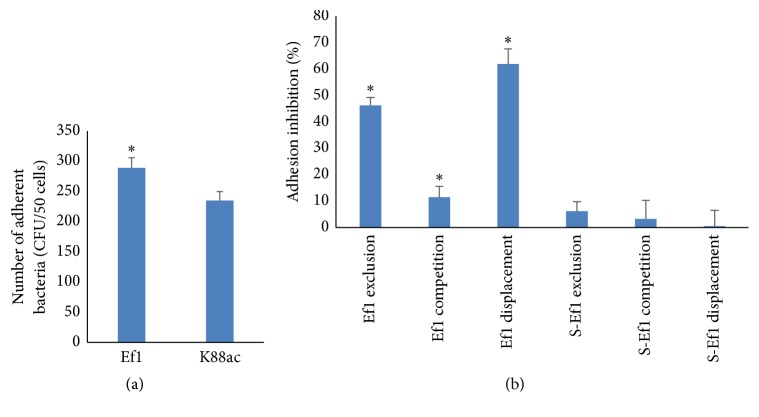
Inhibitory effects of Ef1 and S-Ef1 on K88ac attachment to IPEC-J2 cells. (a) The adhesion of Ef1 and K88ac. Fully differentiated IPEC-J2 cells were treated with 5 × 10^7^ CFU of Ef1 or 5 × 10^7^ CFU of K88ac, respectively, for 1 h. The attached bacteria were counted. (b) The inhibition effect of Ef1 and S-Ef1 on ETEC K88ac adhesion. Ef1-K88ac, Ef1+K88ac, and K88ac-Ef1 represent the inhibition effect of HDRsEf1 to K88ac by exclusion, competition, and displacement, respectively, and S-Ef1-K88ac, S-Ef1+K88ac, and K88ac-S-Ef1 represent the inhibition effect of S-Ef1 on K88ac by exclusion, competition, and displacement, respectively. The K88ac treated alone was used as controls, the columns represent the means ± standard deviation of 3 experiments performed in duplicate, and the presence of various asterisks (*∗*) indicates statistical differences with significant levels of *p* < 0.05.

Ef1 and K88ac were all able to adhere to IPEC-J2 cells after 1 h of incubation, and the adhesion ability of Ef1 is greater than that of K88ac (Figure 1(a)). Coincubation, preincubation, and postincubation of Ef1 with K88ac obviously inhibited the attachment of K88ac, and the greatest inhibition was seen in the replacement group (Figure 1(b)). The Ef1 supernatants did not prevent K88ac adhesion (Figure 1(b)).

### 3.2. Effects of HDRsEf1 and Its Culture Supernatant on the Expression of IL-8 in IPEC-J2 Cells

ETEC, which is a known pathogen and stimulator of IL-8, can damage IECs by modulating cytokines [27, 28]. In order to assess the anti-inflammatory properties of HDRsEf1, IPEC-J2 cells were pretreated with HDRsEf1 or its supernatant for 2 h and then treated with K88ac, TNF-*α*, or IL-1*β*, and the expression of IL-8 was measured by qRT-PCR and ELISA.

#### 3.2.1. Ability of Ef1 and S-Ef1 to Attenuate K88ac-Induced IL-8 mRNA Expression

**Figure 2:**
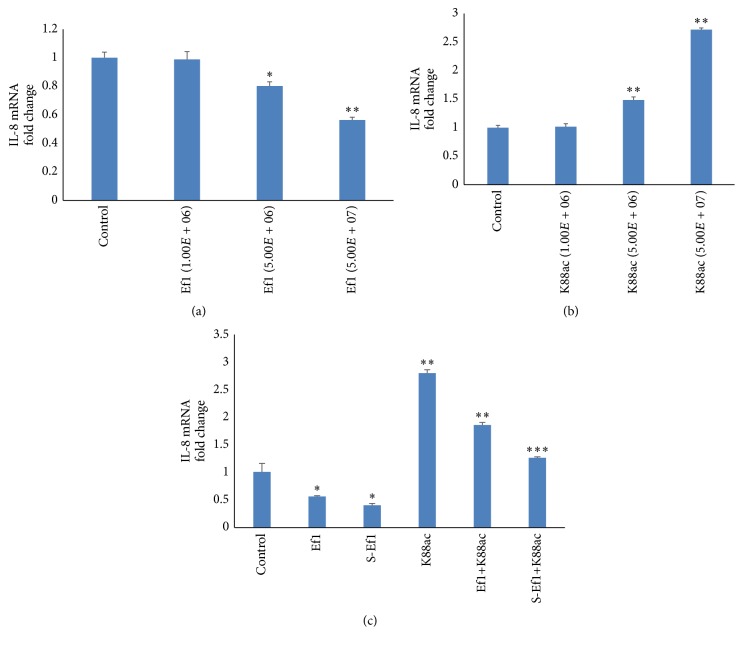
Effects of Ef1 on IL-8 production in IPEC-J2 cells stimulated by K88ac. (a) Three-day cultured IPEC-J2 cells in 100% confluence were stimulated with various concentrations of HDRsEf1 for 2 h and the levels of IL-8 mRNAs were detected using qRT-PCR. (b) Three-day cultured IPEC-J2 cells in 100% confluence were stimulated with various concentrations of K88ac for 2 h and the levels of IL-8 mRNAs were detected using qRT-PCR. (c) Three-day cultured IPEC-J2 cells were incubated with 5 × 10^7^ CFU of Ef1 or S-Ef1 for 2 h and then challenged with 5 × 10^7^ CFU K88ac for 2 h, and the levels of IL-8 mRNAs were detected using qRT-PCR. Untreated IPEC-J2 cells were used as controls, the columns represent the means ± standard deviation of 3 experiments performed in duplicate, and the presence of various asterisks (*∗*, *∗∗*, and *∗∗∗*) indicates statistical differences with significant levels of *p* < 0.05, *p* < 0.01, and *p* < 0.001, respectively.

Firstly, IPEC-J2 cells were stimulated by different concentrations of HDRsEf1 or K88a for 2 h. And it was found that 5 × 10^7^ CFU/mL of HDRsEf1 clearly downregulated the IL-8 mRNA level, while K88ac strongly upregulated it (Figures 2(a) and 2(b)).

Secondly, we investigated the ability of HDRsEf1 and its supernatant to affect the response of IPEC-J2 cells to K88ac. IPEC-J2 cells were challenged with K88ac after treatment with HDRsEf1 or its supernatant. When the IPEC-J2 cells were challenged with K88ac for 2 h, the IL-8 mRNA level increased as much as 3-fold (*p* < 0.001). However, if the IPEC-J2 cells were pretreated by HDRsEf1 or S-Ef1 for 2 h, the IL-8 level was reduced by about one-third (*p* < 0.001) or one-half (*p* < 0.001), respectively (Figure 2(c)). These results indicated that both HDRsEf1 and its secret molecules could significantly inhibit IL-8 expression induced by K88ac, and the later one was stronger inhibitor.

#### 3.2.2. Ability of Ef1 and S-Ef1 to Attenuate IL-1*β*/TNF-*α*-Induced IL-8 mRNA Expression

**Figure 3:**
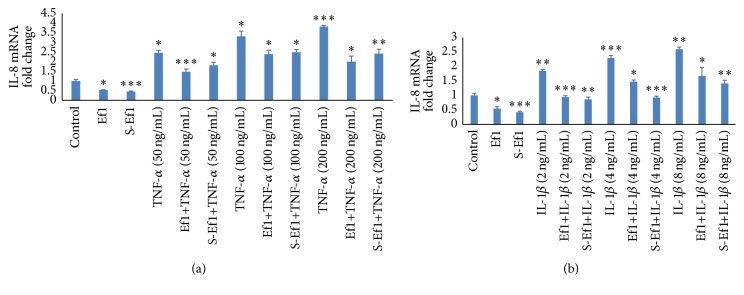
Effects of Ef1 or S-Ef1 on IL-8 mRNA on IPEC-J2 cells stimulated by TNF-*α* /IL-1*β*. (a) Three-day cultured IPEC-J2 cells in 100% confluence were incubated with 5 × 10^7^ CFU Ef1 or S-Ef1 for 2 h and then stimulated with TNF-*α* for 2 h, and the levels of IL-8 mRNAs were detected using qRT-PCR. (b) Three-day cultured IPEC-J2 cells in 100% confluence were incubated with 5 × 10^7^ CFU of Ef1 or S-Ef1 for 2 h and then stimulated with IL-1*β* for 2 h, and the levels of IL-8 mRNAs were detected using qRT-PCR. Untreated IPEC-J2 cells were used as controls, the columns represent the means ± standard deviation of 3 experiments performed in duplicate, and the presence of various asterisks (*∗*, *∗∗*, and *∗∗∗*) indicates statistical differences with significant levels of *p* < 0.05, *p* < 0.01, and *p* < 0.001, respectively.

Some endogenous cytokines can increase the release of IL-8 in IECs and cause severe inflammation. Therefore, we investigated whether HDRsEf1 or its supernatant could prevent IPEC-J2 cells from initiating an inflammatory response. Firstly, IPEC-J2 cells were incubated with HDRsEf1 or its supernatant for 2 h and then treated with various concentration of TNF-*α* or IL-1*β* to mimic an inflammatory context. As shown in Figure 3, TNF-*α* and IL-1*β* stimulation upregulated the IL-8 mRNA level dose-dependently and 200 ng/mL of TNF-*α* and 8 ng/mL of IL-1*β* increased the mRAN of IL-8 approximately 3.8-fold (*p* < 0.001) and 2.6-fold (*p* < 0.001) (Figure 3), respectively. However, HDRsEf1 or S-Ef1 preincubation could downregulate the mRNA of IL-8 in IPEC-J2 cells trigged by TNF-*α* and IL-1*β*. Compared with TNF-*α* (200 ng/mL) and IL-1*β* (8 ng/mL) treatment alone, HDRsEf1 preincubation decreased the mRNA of IL-8 approximately 2-fold (*p* < 0.001) and 1.7-fold (*p* < 0.05), respectively, and S-Ef1 preincubation deceased the mRNA of IL-8 about 2.4-fold (*p* < 0.001) (Figure 3(a)) and 1.4-fold (*p* < 0.001) (Figure 3(b)), respectively.

#### 3.2.3. Ability of Ef1 or S-Ef1 to Attenuate K88ac/IL-1*β*/TNF-*α*-Induced IL-8 Production

**Figure 4:**
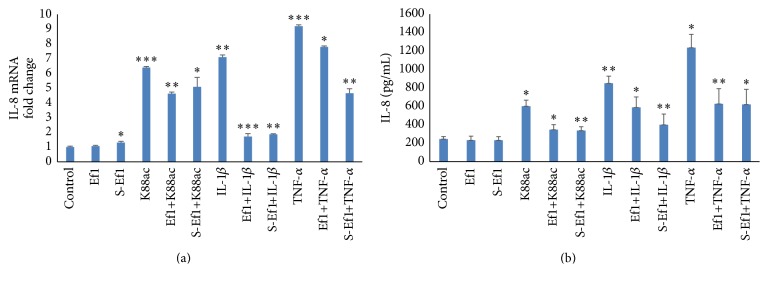
Effects of Ef1 or S-Ef1 on the expression of IL-8 on IPEC-J2 cells stimulated by K88ac/IL-1*β*/TNF-*α*. (a) Three-day cultured IPEC-J2 cells in 100% confluence were incubated with 5 × 10^7^ CFU of Ef1 or S-Ef1 for 2 h and then stimulated with K88ac, TNF-*α* (200 ng/mL), and IL-1*β* (8 ng/mL) for 4 h, and the levels of IL-8 mRNAs were detected using qRT-PCR. (b) Three-day cultured IPEC-J2 cells in 100% confluence were incubated with 5 × 10^7^ CFU of Ef1 or S-Ef1 for 2 h and then stimulated with K88ac, TNF-*α* (200 ng/mL), and IL-1*β* (8 ng/mL) for 4 h, and the proteins of IL-8 were detected by ELISA. Untreated IPEC-J2 cells were used as controls, the columns represent the means ± standard deviation of 3 experiments performed in duplicate, and the presence of various asterisks (*∗*, *∗∗*, and *∗∗∗*) indicates statistical differences with significant levels of *p* < 0.05, *p* < 0.01, and *p* < 0.001, respectively.

In the end, in order to verify whether HDRsEf1 or its supernatant could have a long-term effect of inflammation, we extended the time of stimulation with K88ac (5 × 10^7^ CFU/mL), TNF-*α* (200 ng/mL), or IL-1*β* (8 ng/mL) from 2 h to 4 h and then determined the IL-8 mRNA and protein levels. After 4 h of treatment with K88ac, TNF-*α*, IL-1*β*, the IL-8 mRNA, and protein levels increased significantly (Figure 4); the mRNA level increased by about 6.4 times (*p* < 0.001), 9.2 times (*p* < 0.001), and 7.1 times (*p* < 0.001) (Figure 4(a)), respectively; and the protein level of IL-8 reached about 602 pg/mL (*p* < 0.05), 1237 pg/mL (*p* < 0.01), and 850 pg/mL (*p* < 0.001) versus the control at 244 pg/mL (Figure 4(b)), respectively. However, pretreatment with either HDRsEf1 or S-Ef1 inhibited IL-8 levels in IPEC-J2 cells. With HDRsEf1 preincubation, the mRNA level of IL-8 decreased 4.6-fold (*p* < 0.001), 7.8-fold (*p* < 0.05), and 1.7-fold (*p* < 0.001) (Figure 4(a)) compared with treatment of K88ac, TNF-*α*, and IL-1*β* alone, respectively, and the secretion of IL-8 decreased to about 347 pg/mL (*p* < 0.01), 626 pg/mL (*p* < 0.01), and 589 pg/mL (*p* < 0.05) (Figure 4(b)) versus K88ac, TNF-*α*, and IL-1*β*, respectively. With S-Ef1 preincubation, the mRNA of IL-8 decreased by about 5.1-fold (*p* < 0.05), 4.7-fold (*p* < 0.001), 1.9-fold (*p* < 0.001) (Figure 4(a)), and the secretion of IL-8 decreased to about 3.36 pg/mL (*p* < 0.01), 621 pg/mL (*p* < 0.01), and 400 pg/mL (*p* < 0.01) (Figure 4(b)), compared with treatment of K88ac, TNF-*α*, and IL-1*β* alone, respectively.

### 3.3. The Influence of Heat-Inactivated HDRsEf1 and S-Ef1 on the Expression of IL-8 in IPEC-J2 Cells

**Figure 5:**
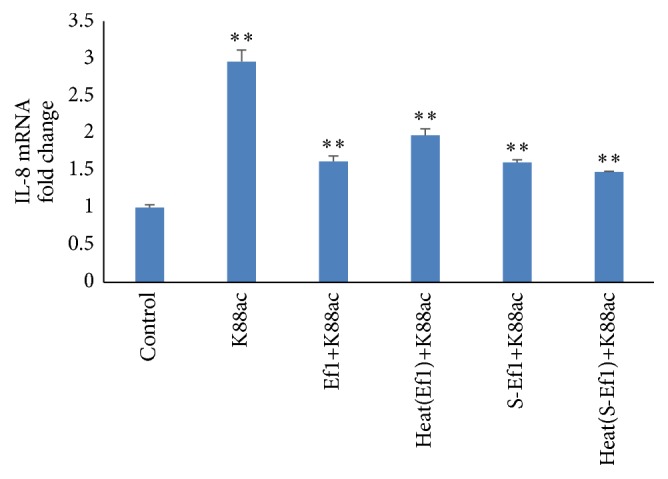
Effects of heat treatment of HDRsEf1 and S-Ef1 on the mRNA of IL-8 in IPEC-J2 cells. Three-day cultured IPEC-J2 cells in 100% confluence were incubated with 5 × 10^7^ CFU of Ef1, S-Ef1, heat-inactivated Ef1, and heat-inactivated S-Ef1 for 2 h and then stimulated by K88ac for 2 h, and the levels of IL-8 mRNAs were detected using qRT-PCR. Results represent means ± standard deviations from three independent experiments. The presence of various asterisks (*∗∗*) indicates statistical differences with significant levels of *p* < 0.01.

Pretreatment with heat-inactivated HDRsEf1 and S-Ef1 reduced the mRNA levels of IL-8 induced by K88ac (*p* < 0.001) and the mRNA levels of IL-8 were similar to that of the live HDRsEf1 and S-Ef1 (*p* > 0.05) (Figure 5). These results showed that heat treatment had no effect on the regulation of inflammation by Ef1 or S-Ef1. The regulatory capacity of HDRsEf1 was related to its cell surface structures, and the anti-inflammatory components from S-Ef1 were insensitive to heat.

### 3.4. Effects of HDRsEf1 on Epithelial Barrier Function

**Figure 6:**
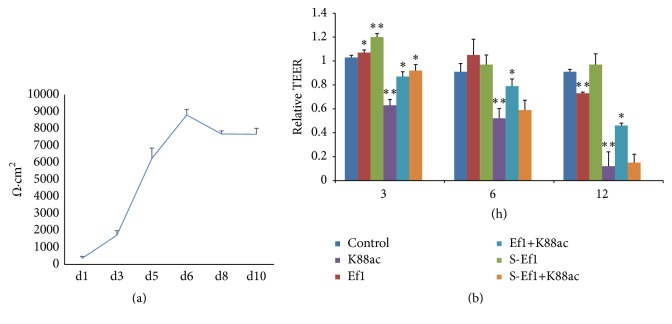
Effects of Ef1 and S-Ef1 on TEER in IPEC-J2 cells. (a) Progression in TEER values of cells grown on Transwell filter for 10 days. (b) Polarised cells were untreated or treated with 5 × 10^7^ CFU of Ef1 or S-Ef1 for 2 h and then stimulated with 5 × 10^7^ CFU of K88ac for 0, 3, 6, and 12 h. The changes of TEER during incubation with bacteria were calculated based on the TEER values of PEC-J2 cells at 0 h. Data given are means (±SEM) of at least four separate experiments. The presence of various asterisks (∗ and ∗∗) indicates statistical differences with significant levels of *p* < 0.05 and *p* < 0.01, respectively.

The effect of HDRsEf1 and its cell-free supernatant on epithelial barrier function was studied by measuring TEER. TEER has been used as an indicator of intestinal barrier integrity [29]. In our study, TEER of IPEC-J2 cells was measured on days 1 and 2 and every other day thereafter. TEER increased dramatically from day 2 to day 6 and then plateaued (Figure 6(a)). When TEER was stable, the IPEC-J2 cells were pretreated with HDRsEf1 or its supernatant (1 × 10^8^ CFU/mL/well) for 2 h and then treated with K88ac (1 × 10^8^ CFU/mL/well). The results showed that HDRsEf1 and S-Ef1 increased TEER at an early stage and that K88ac could significantly disrupt TEER in IPEC-J2. After stimulation with K88ac 3, 6, or 12 hours later, the levels of TEER decreased to 0.63 (*p* < 0.01), 0.52 (*p* < 0.01), or 0.12 (*p* < 0.01) relative to the original (1.0) (Figure 6(b)). However, pretreatment with either HDRsEf1 or S-Ef1 inhibited the decrease in TEER caused by K88ac at an earlier stage (*p* < 0.05). HDRsEf1 had a long-term protective effect: 12 hours later, the epithelial barrier was functional (*p* < 0.05), while, with S- Ef1, the barrier was dysfunctional 3 hours later (Figure 6(b)).

### 3.5. Effect of EPS and Protein from S-Ef1 on IL-8 Expression in IPEC-J2 Cells

**Figure 7:**
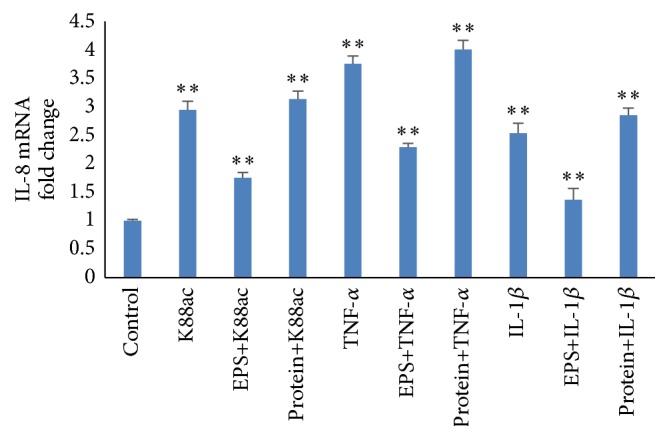
Effects of EPS and protein on the expression of IL-8 in IPEC-J2 cells. Three-day cultured IPEC-J2 cells in 100% confluence were incubated with 5 × 10^7^ CFU of EPS and protein from S-Ef1 for 2 h and then stimulated with K88ac, TNF-*α* (200 ng/mL), and IL-1*β* (8 ng/mL) for 2 h, and the levels of IL-8 mRNAs were detected using qRT-PCR. Results represent means ± standard deviations from three independent experiments. The presence of various asterisks (∗∗) indicates statistical differences with significant levels of *p* < 0.01.

Pretreatment with EPS from S-Ef1 reduced mRNA level of IL-8 induced by K88ac (*p* < 0.001), TNF-*α* (*p* < 0.001), and IL-1*β* (*p* < 0.01) while the protein had no effect (Figure 7). This results showed that EPS could significantly downregulate the expression of IL-8 caused by K88ac.

## 4. Discussion

The aim of this study was to elucidate the effects of the probiotic* Enterococcus faecium* HDRsEf1 or its cell-free supernatant on intestinal epithelial barrier function and inflammatory responses. To examine whether HDRsEf1 could modify the epithelial response to challenge by a pathogen and inflammation mediators, epithelial cell monolayers were incubated with ETEC K88ac, IL-1*β*, or TNF-*α*. Our hypothesis was that epithelial integrity would be enhanced and expression of IL-8 would be reduced due to the action of HDRsEf1.

For enteropathogens, attachment to IECs represents an essential step in establishing an infection. In pigs, ETEC is the most common etiologic agent of enteric diseases in the weaning period. ETEC infection induces a proinflammatory response in porcine IECs [30] and causes diarrhoea that results in reduced growth, mortality, and economic loss [8]. Epithelial adhesion is crucial for this pathogen to colonise an intestine, produce inhibitory compounds, reduce luminal pH, and compete for nutrients [31, 32]. The IPEC-J2 cell line is functionally valid for use in ETEC infection studies [33, 34]. In this study, HDRsEf1 was shown to be effective in inhibiting the adhesion of ETEC K88ac to IPEC-J2 cells; specifically, HDRsEf1 exerted strong displacement activity toward ETEC K88ac. A survey of the literature indicates that the displacement activity exerted by probiotic bacteria toward enteropathogens is related to mechanisms other than mere competition for common adhesion sites [35]. Lievin et al. demonstrated that* Bifidobacterium* strains isolated from infants produce antibacterial lipophilic factor(s) effective in inhibiting* Salmonella enterica* serovar Typhimurium invasion of Caco-2 cells and in killing intracellular enteropathogens [36]. Fujiwara et al. reported that a proteinaceous factor could inhibit* in vitro* adherence of an ETEC strain to gangliotetraosylceramide molecules, which are physiological constituents of the mammalian intestinal epithelial surface [37, 38]. Coconnier et al. demonstrated that the antagonistic activity of LAB against* S. choleraesuis* serovar Typhimurium was due to an antimicrobial compound present in the culture supernatant of LB [39]. In this study, Ef1 supernatant had no effect on the adhesion of ETEC to IPEC-J2 cells, perhaps due to the low concentration of Ef1 supernatant.

Despite the known association between impaired intestinal barrier function, gastrointestinal disorders [40, 41], and diseases in other parts of the body [42, 43], few studies have focused on probiotics that enhance intestinal barrier function. TEER is an index of paracellular and transcellular resistance that has been used to assess epithelial integrity [44, 45]. Studies have shown that some bacteria can enhance intestinal barrier function. One of the proposed mechanisms of probiotic LAB action is strengthening of the epithelial barrier [46, 47]. Therefore, in this study, TEER of the IPEC-J2 cell monolayer was measured. Because ETEC can disrupt barrier integrity, ETEC was used as a control, and, as expected, IPEC-J2 cells preincubated with HDRsEf1 or its supernatant inhibited the decrease in TEER that was caused by ETEC. Thus, HDRsEf1 can fortify intestinal barrier function by tightening the epithelial cell layer junctions.

Further, proinflammatory cytokines can be modulated by the microbiota in the gastrointestinal tract. Symbiotic bacteria, especially probiotic bacteria, can modify the expression of cytokines from epithelial cells [48, 49]. When the gastrointestinal tract is infected by enteropathogenic bacteria, epithelial cells can secrete IL-8 and other proinflammatory factors to fight against foreign substances and to recruit neutrophils and other inflammatory cells. In some cases, a massive and prolonged infiltration of neutrophils may lead to cell damage, epithelial barrier dysfunction, and the pathophysiology of diarrhoea. Altered cytokine release, in turn, can regulate the structure and function of tight junctions and the cytoskeleton [50, 51], as well as the transport properties of epithelial cells [52]. According to our data, HDRsEf1 and its supernatant have ability to protect intestinal cells against an acute inflammatory response. HDRsEf1 and S-Ef1 both were effective in inhibiting IL-8 production in IPEC-J2 cells stimulated by TNF-*α*, IL-1*β*, or K88ac. The results of this study indicated that HDRsEf1 can modify IL-8 levels that are effective against enteropathogens and proinflammatory factors. Our data are in agreement with recent reports [15, 53] that commensal bacteria or probiotics can downregulate IL-8 released by IECs to fight against the enteropathogens and reduce proinflammatory factors. The supernatants of* Lactobacillus rhamnosus* L34 and* L. casei* L39 can inhibit* Clostridium difficile*-induced IL-8 production in IECs [54]. Some reports had elaborated that probiotics and their components could modulate inflammatory responsiveness and TLR-related gene expression [55, 56], such that* L. amylovorus *and its supernatant inhibit TLR4 inflammatory signalling triggered by ETEC, and TLR2 is required for the suppression of TLR4 signalling [27]. EPS of* L. delbrueckii *have been shown to attenuate ETEC-induced inflammatory responses in porcine IECs, with TLR2/TLR4 playing a central role in the immunomodulatory action [57]. Further, Kainulainen et al. [58] showed that EPS of LAB20 might have a role in the immunomodulatory activity of LAB20. Our results indicate that EPS of HDRsEf1 may play a similar role in the immunomodulatory activity of Ef1.

In conclusion, we demonstrated that HDRsEf1 can adhere to IECs and inhibit IEC adhesion and proinflammatory action of ETEC K88ac. Specifically, it can fortify the epithelial cell layer and elicit anti-inflammatory responses in enterocytes. It is EPS rather than proteins in Ef1 cultural supernatant that do the probiotic effect, but the precise mechanisms of and the exact components of EPS that contribute to anti-inflammatory functions remain to be identified.

## Figures and Tables

**Table 1:** Primers for qRT-PCR.

Primer name	Sequence	Amplicon size (bp)
IL-8-F	AGAACTTCGATGCCAGTGC	143 bp
IL-8-R	GGCAGACCTCTTTTCCATTG	
*β*-actin-F	CATCACCATCGGCAACGA	144 bp
*β*-actin-R	GCGTAGAGGTCCTTCCTGATGT	[23]
